# Identifying Selection Signatures for Backfat Thickness in Yorkshire Pigs Highlights New Regions Affecting Fat Metabolism

**DOI:** 10.3390/genes10040254

**Published:** 2019-03-28

**Authors:** Haoran Ma, Saixian Zhang, Kaili Zhang, Huiwen Zhan, Xia Peng, Shengsong Xie, Xinyun Li, Shuhong Zhao, Yunlong Ma

**Affiliations:** Key Laboratory of Agricultural Animal Genetics, Breeding, and Reproduction of the Ministry of Education & Key Laboratory of Swine Genetics and Breeding of the Ministry of Agriculture, Huazhong Agricultural University, Wuhan 430070, China; 17671710421@163.com (H.M.); zhangsaixian@163.com (S.Z.); zkl1153793935@163.com (K.Z.); wenhuizhan412@gmail.com (H.Z.); pengxia418@163.com (X.P.); ssxie@mail.hzau.edu.cn (S.X.); xyli@mail.hzau.edu.cn (X.L.); shzhao@mail.hzau.edu.cn (S.Z.)

**Keywords:** trait-specific selection signatures, population differentiation-based methods, functional annotation

## Abstract

Identifying the genetic basis of improvement in pigs contributes to our understanding of the role of artificial selection in shaping the genome. Here we employed the Cross Population Extended Haplotype Homozogysity (XPEHH) and the Wright’s fixation index (F_ST_) methods to detect trait-specific selection signatures by making phenotypic gradient differential population pairs, and then attempted to map functional genes of six backfat thickness traits in Yorkshire pigs. The results indicate that a total of 283 and 466 single nucleotide polymorphisms (SNPs) were identified as trait-specific selection signatures using F_ST_ and XPEHH, respectively. Functional annotation suggested that the genes overlapping with the trait-specific selection signatures such as *OSBPL8*, *ASAH2*, *SMCO2*, *GBE1,* and *ABL1* are responsible for the phenotypes including fat metabolism, lean body mass and fat deposition, and transport in mouse. Overall, the study developed the methods of gene mapping on the basis of identification of selection signatures. The candidate genes putatively associated with backfat thickness traits can provide important references and fundamental information for future pig-breeding programs.

## 1. Introduction

In past decades, high-intensity artificial selection has been applied to the genetic improvement of commercial traits in pigs, including backfat thickness, lean meat content, growth, and feed conversion efficiency [1]. Backfat thickness is an important breeding objective trait, because it indirectly affects carcass lean percentage, fat deposition, meat quality, and consumers’ acceptance of pork [2]. Although many genome wide association (GWA) studies have been conducted in the past decades with the development of molecular marker technology, the genetic mechanism affecting backfat thickness still appears to be confusing [2,3,4,5]. From the view of population genetics, the effect of artificial selection affecting backfat thickness would leave detectable selection signatures within the pig genome [6,7,8]. Therefore, identifying the selection signatures underlying these phenotypic changes provides new insight into general mechanisms by which genetic variation shapes the backfat thickness traits.

In this study, we attempted to detect genomic selection signatures for backfat thickness traits in Yorkshire pigs. However, the genomic region revealed by the identification of selection signatures is always very broad-spectrum, containing functional candidate genes associated with many traits. To map the genes for a single specific trait, we often need to infer the corresponding genomic regions in conjunction with the results of bioinformatics analysis, such as enrichment analysis, pathway analysis, the Quantitative Trait Loci (QTL) annotation, and comparative genomic analysis [7,9,10]. As a complex trait, the backfat thickness is controlled by polygenes and the effect of each gene is small. Therefore, in this study, we put forward an important hypothesis that most of the loci associated with backfat thickness are still polymorphic in spite of the fact that corresponding allele frequencies are improved by high-intensity artificial selection.

So, we could make full use of the inter-population selection signatures detection methods to map the genes associated with the backfat traits through constructing the phenotypic gradient differential population pairs. This study developed the methods of gene mapping on the basis of the identification of selection signatures. The results would provide useful information for those who are interested in further understanding the genetic basis of backfat thickness and facilitate the future breeding of pigs to improve this trait through molecular marker-assisted selection.

## 2. Materials and Methods

### 2.1. Ethics Approval

All research involving animals was conducted under protocols (No. 5 proclaim of the Standing Committee of Hubei People’s Congress) approved by the Standing Committee of Hubei People’s Congress and the ethics committee of Huazhong Agricultural University in China. All experiments were performed in accordance with approved relevant guidelines and regulations.

### 2.2. Animals and Phenotype

In this study, a total of 233 castrated Yorkshire pigs were raised under the same conditions and slaughtered at a weight of 90 kg. The backfat thickness traits that did not include the skin thickness were measured on the right half of the carcass using a vernier caliper. The backfat thickness traits include: (i) Shoulder subcutaneous fat thickness (Represented by BF1); (ii) backfat between 6th and 7th thoracic vertebras (Represented by BF2); (iii) backfat at 10th rib (Represented by BF3); (iv) backfat at thoracolumbar junction (Represented by BF4); (v) backfat at waist recommended vertebral junction (Represented by BF5); and (vi) the average backfat thickness of shoulder subcutaneous fat thickness, backfat at thoracolumbar junction and backfat at waist recommended vertebral junction (Represented by BF6).

### 2.3. Genotype and Quality Control

Genomic DNA of each individual was extracted from ear tissues using a standard phenol-chloroform method and diluted to 50 ng/mL. All 233 individuals were genotyped together using Illumina PorcineSNP60 BeadChips (Illumina, San Diego, CA, USA), which includes 62,163 SNPs (single nucleotide polymorphisms) across the entire pig genome. Quality control of SNP data applied the following criteria: (i) individual call rate > 0.90; (ii) SNP call rate > 0.95; (iii) SNPs in Hardy–Weinberg equilibrium in each breed (*p* > 1 × 10^−6^); (iv) SNP minor allele frequency > 0.05; and (v) only autosomal SNPs with known positions were used. After quality control, we imputed the missing genotypes and inferred haplotypes using BEAGLE (Version 3.3.2) [11].

### 2.4. Linkage Disequilibrium, Allele Frequency and Heterozygosity

We calculated the correlation coefficient (*r*^2^) for every pair of SNPs to measure the linkage disequilibrium (LD) level in Yorkshire pigs using PLINK (Version 1.90) software [12]. To visualize the LD decay, the *r*^2^ values for 1 kb distance bins were averaged and the corresponding figures were drawn by R script. To further assess the genomic characteristics of Yorkshire pigs, the minor allele frequency and the heterozygosity of each SNP were also calculated in this analysis.

### 2.5. Detecting Postive Selection Signatures by iHS

In this analysis, the integrated Haplotype Homozygosity Score (iHS) was used to detect positive selection signatures. Single marker scores for unstandardized iHS were calculated using the rehh R package and then the |iHS| scores averaged across a non-overlapping 50 kb window across the genome [13,14]. The empirical *p*-values were generated by genome-wide ranking of |iHS| scores.

### 2.6. Identification of Trait-Specific Selection Signatures

To further detect the trait-specific selection signatures, this study first produced three phenotypic gradient differential population pairs. Taking the shoulder subcutaneous fat thickness (Represented by BF1) trait as an example, the basic steps are as follows: (i) ranking the phenotypic value; (ii) according to the ranking result, the population was divided into two subpopulations: high backfat thickness and low backfat thickness, which were recorded as the 1st population pair; (iii) based on the second step, 75 individuals with thicker backfat thickness were selected from the high backfat thickness subpopulation, and 75 individuals with thinner backfat thickness were selected from the low backfat thickness subpopulation to create a phenotypic gradient differential population pair, recorded as the 2nd population pair; (iv) based on the third step, 45 individuals with thicker backfat thickness were selected from 75 individuals with thick backfat thickness, and 45 individuals with thinner backfat thickness were selected from 75 individuals with thin backfat thickness to create a phenotypic gradient differential population pair, recorded as the 3rd population pair; (v) the inter-population methods were used to detect selection signatures in the three phenotypic gradient differential population pairs, respectively. The other backfat thickness traits were performed following the same procedure.

The Cross Population Extended Haplotype Homozogysity (XPEHH) [15] and the Wright’s fixation index (F_ST_) [16] were used to detect selection signatures in the three phenotypic gradient differential population pairs for each trait. The script of XPEHH is available at http://hgdp.uchicago.edu. In general, a negative XPEHH score suggests that selection happened in the reference population, whereas selection happened in the observed population [15]. The XPEHH scores did not need to be normalized in this analysis. The XPEHH method has a high power to detect selection signatures with almost or fully fixed haplotypes [7]. The F_ST_ method proposed by Weir and Cockerham was used to measure the population differentiation, with values ranging from 0 (no differentiation) to 1 (complete differentiation) [16]. This method is sensitive in detecting fixed selection signatures [7].

In this study, the empirical *p*-values were generated by genome-wide ranking of F_ST_ and XPEHH values [8,17]. The selection signature that fulfilled the following criteria was defined as a trait-specific selection signature. The criteria included: (i) the *p* value of the statistic reached a significant level of 0.01 in the three phenotypic gradient differential population pairs for each trait; (ii) From 1st to 3rd population pair, the significant SNPs in which the F_ST_ and XPEHH statistic values exhibited a gradient change were defined as the backfat traits’ specific selection signatures.

### 2.7. Functional Annotation for Trait-Specific Selection Signatures

To explore the biological functions of genes overlapping with the trait-specific selection signatures, the genomic regions within 200 kb distance bin around the trait-specific selection signatures were defined as trait-specific selection regions. Genes located in the putative selection regions were identified using the BioMart program (http://www.biomart.org/) referring to the database of “Ensembl Genes 95” and “Pig genes (Sscrofa 11.1)”, and then the orthologous comparison analysis was performed for the identified genes function on the basis of the international database of Mouse Genome Informatics (MGI) [18]. The enrichment analysis was performed for the list of genes located in putative selection regions using DAVID 6.8 (https://david.ncifcrf.gov/) [19]. Accordingly, the terms that had *p* values less than 0.05 were considered as significant after Bonferroni correction. We functionally annotated trait-specific selection signatures with the gene-based annotation modules of ANNOVAR [20]. Using ENSEMBL genes, we investigated where the SNPs are located in the regions of gene components. To further explore the biological function of trait-specific selection signatures, the QTLs enrolled in Pig QTLdb (www.animalgenome.org) were gathered and compared with those candidate selection regions based on the putative location of the QTLs.

## 3. Results

### 3.1. Qualified SNPs and Animals in the Analysis

In this analysis, a total of 233 performance-tested Yorkshire pigs were genotyped with the Illumina PorcineSNP60 BeadChip. The SNPs were re-mapped on the Sscrofa11.1 genome assembly according to the position information published by Illumina and SNPdb. After quality control, 11,624 SNPs that had a minor allele frequency of <0.05 were discarded, 42 markers to be excluded based on HW test (*p* ≤ 1 × 10^−6^). The final dataset consisted of 37,061 SNPs at 18 autosomes with an average inter-marker spacing of 62.06 kb. Among them, a total of 5446 SNPs that passed quality control but still had a SNP missing rate of less than 0.05 were imputed by Beagle.

### 3.2. The summary of Phenotype

**Figure 1:**
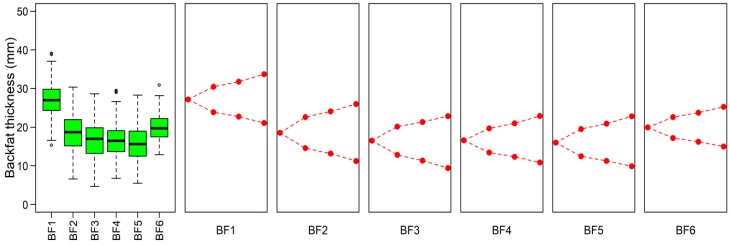
Descriptive statistics of six backfat thickness traits and the visualization of three phenotypic gradient differential population pairs for each trait. The left ‘box-and-whiskers’ plot describes the distribution of a continuous phenotypic variable. The other plots describe three phenotypic gradient differential population pairs for each trait. In order from left to right, the source Yorkshire population, the 1st population pair, the 2nd population pair and the 3rd population pair are stretched along each drawing area, and the phenotypic differentiation is gradually increased. BF1: Shoulder subcutaneous fat thickness; BF2: backfat between 6th and 7th thoracic vertebras; BF3: backfat at 10th rib; BF4: backfat at thoracolumbar junction; BF5: backfat at waist recommended vertebral junction; BF6: the average backfat thickness of shoulder subcutaneous fat thickness, backfat at thoracolumbar junction and backfat at waist recommended vertebral junction.

Figure 1 displays descriptive statistics of six backfat thickness traits in Yorkshire pigs. As shown in Table S1, all six traits are significantly and positively correlated with each other (*p* < 0.001). The 27.16-mm thickness of shoulder subcutaneous fat is the largest on average in all six traits. The smallest one is the thickness of backfat at the 10th rib, which is about 16.51 mm. As described above, the 233 Yorkshire pigs were divided into three phenotypic gradient differential population pairs according to the phenotypic value for each trait. From the 1st population pair to the 2nd population pair to the 3rd population pair, the average phenotypic value in high backfat thickness is thicker and thicker and the average phenotypic value in low backfat thickness is thinner and thinner. The average phenotypic value of population pairs for all traits are significant differences (*p* < 0.001).

### 3.3. Genomic Characters among Three Phenotypic Gradient Differential Population Pairs

**Figure 2:**
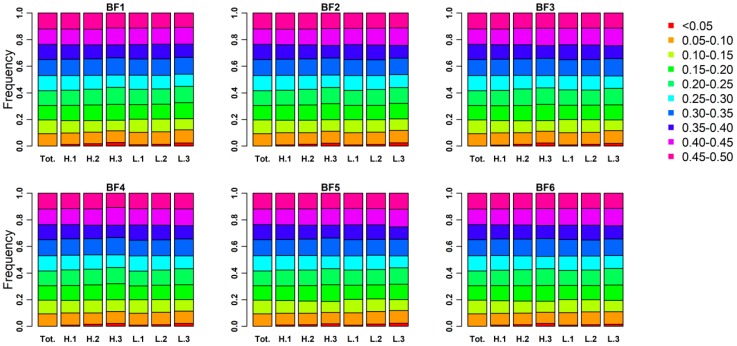
The distributions of minor allele frequency in each subpopulation for backfat thickness traits. Rectangles of different colors indicate different clusters of MAF (minor allele frequency), while rectangles of different lengths indicate the proportions of markers within different clusters. Tot. represents the source Yorkshire population, H.1 (H.2, H.3) and L.1 (L.2, L.3) represent the two subpopulations of 1st (2nd and 3rd) population pair.

**Figure 3:**
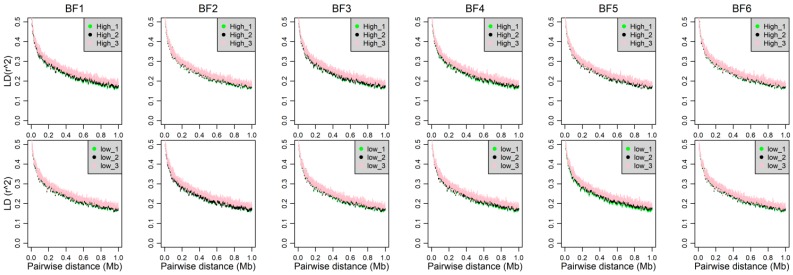
The decay of linkage disequilibrium of all subpopulations across the genome. The pairwise linkage disequilibrium (*r*^2^) is plotted against the corresponding physical distances. High_1 (High_2, High_3) and Low_1 (Low_2, Low_3) represent the two subpopulations of 1st (2nd and 3rd) population pair. LD: linkage disequilibrium.

We investigated the genomic characters of three phenotypic gradient differential population pairs, which can be used to confirm that the selection signatures revealed in this study are trait-specific, rather than differences in accidental genomic patterns caused by population division. As shown in Figure 2, the distributions of minor allele frequency (MAF) estimated from the whole-genome SNPs in each subpopulation are similar with it in the source Yorkshire population. A little increased proportion of MAF between 0 to 0.05 was observed with the decreasing of subpopulation sample size for each trait. In addition, the distributions of expected heterozygosity (He) in each subpopulation are also similar to the source Yorkshire population (Figure S1). To test the degree to which the differences in the LD-decay pattern are caused by population division, we examined LD in all subpopulations. As shown in Figure 3, LD curve decays at a similar rate in all subpopulations. Overall, there is little difference in genomic characters among three phenotypic gradient differential population pairs for each trait. It suggests that the influence from population division will not affect the identification of trait-specific selection signatures.

### 3.4. The Positive Selection Signatures

In this study, the unstandardized iHS scores were calculated per site and the absolute scores were averaged across non-overlapping sliding 50 kb windows across the whole genome. To explore ongoing selection signatures, the windows in which the |iHS| scores fell into the 99th percentile were considered as significant. Out of 44,559 sliding windows, 445 windows were identified as the positive selection signatures in Yorkshire pigs. Then, the windows within a 200 kb fragment around the potential selection signatures were merged and the genomic regions were defined as potential selection regions. Correspondingly, a total of 288 genomic fragments, spanning lengths of 87.35 Mb and covering 3.64% of the genome, were identified as potential selection regions in Yorkshire pigs. Functional annotation suggested that a set of selection signatures with extreme *p*-values coincide with a cluster of genes involved in pigmentation, fat metabolism, fertility, and so on (Table S2). We noted that the genes overlapping with the positive selection signatures are broad-spectrum, which are associated with many important economic traits.

### 3.5. Trait-Specific Selection Signatures

**Figure 4:**
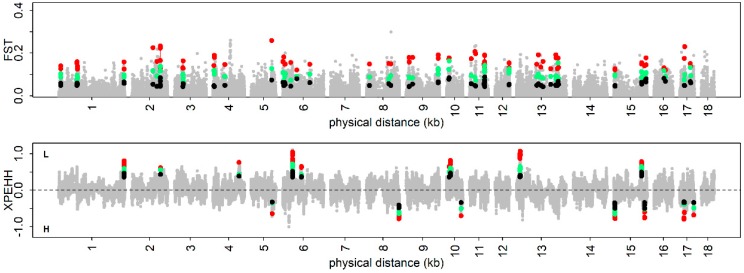
Visualization of trait-specific selection signatures for shoulder subcutaneous fat thickness (BF1). The colored dots indicate the trait-specific selection signatures. The black (green, red) dots indicate the selection signatures identified in 1st (2nd and 3rd) population pair. XPEHH: the Cross Population Extended Haplotype Homozogysity, F_ST_: the Wright’s fixation index.

To identify the trait-specific selection signatures, F_ST_ and XPEHH were used to reveal selection signatures caused by population differentiation. The top 1% of selection signatures were considered as significant and then about 370 SNPs in the three phenotypic gradient differential population pairs for each trait were used for further analysis. Only the common significant SNPs in which the F_ST_ and XPEHH statistic values exhibit a gradient change were defined as the trait-specific selection signatures in this analysis. Using the F_ST_ method, a total of 94 SNPs in BF1, 38 SNPs in BF2, 29 SNPs in BF3, 47 SNPs in BF4, 24 SNPs in BF5, and 51 SNPs in BF6 were identified as trait-specific selection signatures. We functionally annotated trait-specific selection signatures with the gene-based annotation modules of ANNOVAR. Taking BF1 as an example, we identified a total of 94 SNPs as trait-specific selection signatures, of which 65 were intergenic, 26 were intronic and 2 were exonic (Table S3). Similarly, a total of 126 SNPs in BF1, 75 SNPs in BF2, 50 SNPs in BF3, 128 SNPs in BF4, 27 SNPs in BF5, and 60 SNPs in BF6 were identified as trait-specific selection signatures using the XPEHH method (Figure 4 and Figures S2–S6; Table S4). As shown Table S3, we identified a total of 126 SNPs as trait-specific selection signatures in BF1, of which 75 were intergenic, 49 were intronic and 1 was exonic. In this analysis, the adjacent candidate selection signatures within a 400 kb genomic region were combined together. We found that a total of seven genomic regions in BF1, two genomic regions in BF2, two genomic regions in BF3, and two genomic regions in BF6 were detected by two methods simultaneously (Figure 4 and Figures S2–S6).

### 3.6. Candidate Genes at the Identified Loci

Based on the trait-specific selection signatures, the genes that are close to the putative SNPs were picked out according to the available annotation of the pig genome. The enrichment analysis suggested that no significant terms show any intuitive information on selection after correction for multiple testing (Table S5). However, we found that many genes in our list are related with fat metabolism through functional annotation on basis of the MGI database (Table 1). For example, the *OSBPL8* gene was found under selection in the low backfat thickness population of BF4 and its orthologous gene in mouse is associated with the phenotype of “increased circulating triglyceride level” [21]. On the contrary, the ENSSSCG00000039018 gene was found under selection in the high backfat thickness population of BF6 and its orthologous gene (*Tmem167*) in mouse is associated with the phenotype of “decreased circulating triglyceride level”. The ENSSSCG00000010432 gene overlapping with the trait-specific selection signatures of BF2 was found to be orthologous with the *ASAH2* gene, which is related with the phenotype of “abnormal lipid level” in mouse [22]. Note that the *SMCO2* gene detected in the low backfat thickness population of BF4 was associated with the phenotype of “increased lean body mass” [11]. In the process of pig breeding, the backfat thickness traits have always been used as target traits for the cultivation of lean pig breeds [3]. Table S6 summarized the QTLs located in or overlapped with the selection regions identified by XPEHH and F_ST_ simultaneously. Taking the 40436890–41163330 bp selection region of SSC6 as an example, seven QTLs influencing backfat thickness and meat quality were mapped in this region. Simultaneously, the 10868180–11462923 bp selection region of SSC10 and the 57219422–57749302 bp selection region of SSC12 were overlapped with the QTLs associated with fat deposition. Note that many QTLs overlapping with the trait-specific selection regions were related with meat quality traits.

## 4. Discussion

In this study, we attempted to use the inter-population selection signatures method to detect trait-specific selection signatures by making phenotypic gradient differential population pairs, and then map functional genes of the backfat thickness traits. In theory, the backfat thickness traits of pig are a quantitative trait, and their genetic mechanism is complex and controlled by many genes with small effects. This quantitative genetics view is supported by most of the recent gene mapping researches, including genome-wide association studies [3,4,5]. Although these loci are associated with complex traits but are less effect and are not easily detected by genome-wide association analysis, they may be accurately mapped by sweep analysis because they have always been subjected to high-intensity artificial selection. Therefore, it is feasible for the identification of trait-specific selection signatures using inter-population methods through making phenotypic gradient differential population pairs. The results of functional annotation proved this hypothesis, where we found that a series of genes overlapping with trait-specific selection signatures were associated with fat metabolism and deposition (Table 1) [18]. However, there may be some loci that are fixed during the selection process. Therefore, some genes that are indeed selected may not be detected after the quality control of the minimum allele frequencies.

As shown in Table 1, the genes under selection are involved in most of the important biological processes of fat metabolism, transport and fat deposition. For example, the orthologous genes of *GBE1*, *ABL1*, and ENSSSCG00000015039 are related with the phenotype of “liver/biliary system” and “abnormal liver morphology” in mice [23,24]. We also found that some genes identified in this analysis, such as *SEMA6A* and *AZI2*, are related with the nervous system development [25]. It is consistent with the results reported in previous GWAS research, in which the genes associated with the backfat thickness traits of Yorkshire pigs were significantly enriched in the biological processes of nervous system development [2]. Through QTLs annotation, we found that the trait-specific selection signatures mainly overlap with the QTLs of backfat thickness traits and meat quality traits, and even the QTLs of meat quality traits are slightly more (Table S6). This phenomenon indicated that the artificial selection of backfat thickness traits in pigs can have a significant impact on meat quality traits, which is supported by many recent reports [26,27]. In general, we thought there is a significant negative correlation between backfat thickness traits and meat quality traits.

Since the XPEHH method was able to identify the population in which selection occurred, this study was able to further investigate the complex genetic mechanism of backfat thickness traits [15]. In the past decades, the selection direction of pig backfat traits is the process of continuous thinning [3]. However, it does not mean that the selection signatures were always detected in the thin backfat thickness subpopulation. Taking shoulder subcutaneous fat thickness as an example, the number of selection signatures identified in the low backfat thickness subpopulation was 73, but 53 selection signatures were still detected in the high backfat thickness subpopulation (Figure 2; Table S7). Note that the *OSBPL8* gene and the ENSSSCG00000039018 gene were separately identified in low and high backfat thickness subpopulations, while their orthologous genes also have the opposite biological phenotype in mice (Table 1) [11,21]. The results further indicated that the genetic mechanisms of backfat thickness traits are complex. The original purpose of backfat thickness determination is to be used for the cultivation of lean pigs. However, the physiological and biochemical processes involved in backfat deposition are extremely complex, including glycolipid metabolism, fat deposition and transport, and hepatobiliary metabolism. Therefore, it can be inferred that extended phenotypic omics research is an important path for the future analysis of genetic mechanisms of complex traits in pig.

## 5. Conclusions

In this study, a new strategy for the identification of trait-specific selection signatures was presented by making phenotypic gradient differential population pairs and using inter-population selection signature methods. The application for mapping functional genes of six backfat thickness traits in Yorkshire pigs suggested that this strategy is effective against mapping commercial important genes. Simultaneously, this study detected signatures of artificial selection and identified a number of loci associated with backfat thickness traits, like *OSBPL8*, *ASAH2*, *GBE1*, and *ABL1*, which provided an important resource in future pig-breeding programs.

## Figures and Tables

**Table 1:** Summary of results from functional annotation analysis of the trait-specific selection signatures based on the international database of Mouse Genome Informatics (MGI) [18].

Chr.	Position (bp) ^1^	XPEHH (F_ST_) Scores ^2^	*p*-Values_|iHS|_	Genes ^3^	Trait	MGI Phenotype
5	39545743–39744655	0.28 < 0.39 < 0.48	-	*OSBPL8*	Low, BF4	MP:0001552_increased circulating triglyceride level
13	158574324–158574542	−0.31 > −0.34 > −0.57	-	*ENSSSCG00000039018* (*Tmem167*)	High, BF6	MP:0002644_decreased circulating triglyceride level
7	114035383–114541592	0.29 < 0.38 < 0.69	-	*RIN3*	Low, BF4	MP:0005560_decreased circulating glucose level
17	22635204–22723444	−0.32 > −0.37 > −0.64	-	*ENSSSCG00000030112* (*Macrod2*)	High, BF2	MP:0013279_increased fasted circulating glucose level
5	46244283–46274049	0.32 < 0.37 < 0.54	0.005	*SMCO2*	Low, BF4	MP:0003960_increased lean body mass
1	270911878–270912282	0.37 < 0.50 < 0.68	0.025	*QRFP*	Low, BF1	MP:0005375_adipose tissue phenotype MP:0001363_increased anxiety-related response
14	99025306–99129155	(0.03 < 0.06 < 0.11)	0.002	*ENSSSCG00000010432* (*ASAH2*)	-, BF4	MP:0001547_abnormal lipid level
9	39864135–39916486	(0.03 < 0.07 < 0.13)	0.005	*ENSSSCG00000015039* (*BCO2*)	-, BF2	MP:0000003_abnormal adipose tissue morphology
1	270761674–270906709	0.36 < 0.49 < 0.67	0.025	*ABL1*	Low, BF1	MP:0000598_abnormal liver morphology MP:0000607_abnormal hepatocyte morphology
13	173634661–173910739	−0.34 > −0.47 > −0.50, −0.31 > −0.44 > −0.80	0.032	*GBE1*	High, BF3, BF6	MP:0000255_vasculature congestion MP:0005370_liver/biliary system phenotype
1	26473935–26489776	0.33 < 0.50 < 0.13, 0.31 < 0.36 < 0.98	0.002	*TNFAIP3*	Low, BF5, BF6	MP:0001845_abnormal inflammatory response
13	158665077–158729282	−0.31 > -0.34 > −0.57	-	*TBC1D23*	High, BF6	MP:0001846_increased inflammatory response
17	58998981–59055340	−0.34 > −0.48 > −0.68	0.006	*GNAS*	High, BF1	MP:0001845_abnormal inflammatory response
13	175980910–176479481	−0.30 > −0.40 > −0.56	0.032	*ROBO1;*	High, BF6	MP:0005381_digestive/alimentary phenotype
13	14754011–14785502	0.38 < 0.58 < 0.95	-	*AZI2*	Low, BF1	MP:0002376_abnormal dendritic cell physiology
2	120559078–120687331	0.44 < 0.56 < 0.62	0.021	*SEMA6A*	Low, BF1	MP:0000783_abnormal forebrain morphology

^1^ This column presents the position of candidate genes which overlap with or close to the trait-specific selection signatures. ^2^ This column presents the values of sweep statistics that are in three phenotypic gradient change population pairs. ^3^ The gene in brackets is the ortholog of the mouse. hIS: integrated Haplotype Homozygosity Score.

## Supplementary Materials

The following are available online at https://www.mdpi.com/2073-4425/10/4/254/s1, Table S1: the correlation coefficients and the other descriptive statistics of six backfat thickness traits. Table S2: a list of candidate regions revealed by iHS method. Table S3: summary and annotation of the SNPs associated with backfat thickness in Yorkshire pigs. Table S4: the summary of selection signatures for six backfat thickness traits. Table S5: the results of GO analysis and pathway analysis. Table S6: QTLs overlapped with the trait-specific selection signatures identified by F_ST_ and XPEHH simultaneously. Table S7: the summary of XPEHH scores for trait-specific selection signatures. Figure S1: The violin plot of expected heterozygosity in each subpopulation for backfat thickness traits. Figure S2: Visualization of trait-specific selection signatures for backfat between 6th and 7th thoracic vertebras (BF2). Figure S3: Visualization of trait-specific selection signatures for backfat at 10th rib (BF3). Figure S4: Visualization of trait-specific selection signatures for backfat at thoracolumbar junction (BF4). Figure S5: Visualization of trait-specific selection signatures for backfat at waist recommended vertebral junction (BF5). Figure S6: Visualization of trait-specific selection signatures for the average backfat thickness of three points (BF6).
