# Supplementary material for: Identifying Selection Signatures for Backfat Thickness in Yorkshire Pigs Highlights New Regions Affecting Fat Metabolism
Source: Genes (Basel). 2019 Mar 28;10(4):254. doi: 10.3390/genes10040254 (PMC6523431; doi:10.3390/genes10040254)
Supplement: Supplementary file 1 [file genes-10-00254-s001.zip › Supplementary Materials.pdf]

Table S1 the correlation coefficients and the other descriptive statistics of six backfat thickness traits.

	**BF1**	**BF2**	**BF3**	**BF4**	**BF5**	**BF6**
**BF1**	27.16±4.12 mm	<0.001	<0.001	<0.001	<0.001	<0.001
**BF2**	0.51	18.58±4.85 mm	<0.001	<0.001	<0.001	<0.001
**BF3**	0.54	0.77	16.51±4.46 mm	<0.001	<0.001	<0.001
**BF4**	0.46	0.64	0.70	16.56±3.96 mm	<0.001	<0.001
**BF5**	0.47	0.53	0.56	0.55	16.00±4.31 mm	<0.001
**BF6**	0.79	0.69	0.74	0.82	0.84	19.91±3.36 mm

Note: The lower and upper triangle is the correlation coefficient and its p-value, respectively. The diagonal is the mean and standard deviation of all individual phenotypes of the Yorkshire pigs.

Table S2 a list of candidate regions revealed by iHS method.

**Chr.**	**Position (bp)**	***p*_value**	**Genes**	**Function**
1	74139330-74181165	0.007	*OSTM1*	Osteoclast Maturation [1]
2	89855166-89905197	<0.001	*ACOT12*	Fat metabolism [2]
4	93218072-93234226	0.003	*NTRK1*	Nerve Growth Factor [3]
4	33811284-33843294	0.006	*FZD6*	Hair development [4]
6	77161479-77267417	0.007	*PAX7*	Skeletal muscles [5]
7	30320414-30329408	0.008	*HMGA1*	Growth and fat deposition traits [6]
7	64140697-64375535	0.010	*RALGAPA1*	Skeletal muscle [7]
8	102213138-102266309	0.009	*BBS7*	Embryonic development [8]
9	41067491-41135271	0.004	*DRD2*	Pigmentation [9], Fertility [10]
9	89341141-89450696	0.004	*ITGB8*	Pregnant gilts [11]

Table S3 summary and annotation of the SNPs associated with backfat thickness in Yorkshire pigs.

Category	BF1	BF2	BF3	BF4	BF5	BF6
Method	XPEHH (F_{ST})	XPEHH (F_{ST})	XPEHH (F_{ST})	XPEHH (F_{ST})	XPEHH (F_{ST})	XPEHH (F_{ST})
**Number of outliers**	126 (94)	75 (38)	50 (29)	128 (47)	27 (24)	60 (51)
Upstream	-	-	-	-	0 (1)	-
UTR5	-	-	-	-	-	-
Exonic	1 (2)	0 (1)	0 (1)	2 (2)	0 (3)	0 (4)
Intronic	49 (26)	20 (12)	4 (9)	51 (16)	10 (9)	19 (21)
UTR3	1 (1)	2 (0)	-	2 (1)	-	-
Downstream	-	-	-	-	-	0 (1)
Intergenic	75 (65)	53 (25)	46 (19)	73 (28)	17 (11)	41 (25)

Table S4 the summary of significant selection signatures for six backfat thickness traits.

		BF1	BF2	BF3	BF4	BF5	BF6
**1st population pair**	XPEHH;	371;	369;	369;	370;	370;	370;
	F_{ST}	371	371	370	370	370	370
**2nd population pair**	XPEHH;	371;	369;	369;	370;	370;	370;
	F_{ST}	370	371	371	372	372	371
**3rd population pair**	XPEHH;	370;	370;	370;	371;	370;	370;
	F_{ST}	374	370	372	376	370	371
**Common Selection Signatures[2]**	XPEHH;	129;	108;	71;	132;	37;	65;
	F_{ST}	95	42	30	48	27	54
**Trait-specific Selection Signatures[3]**	XPEHH;	126;	75;	50;	128;	27;	60;
	F_{ST}	94	38	29	47	24	51

Table S5 the results of GO analysis and pathway analysis.

See Supplementary Table S5.xlsx

Table S6 QTLs overlapped with the trait-specific selection signatures identified by F_{ST} and XPEHH simultaneously.

Chr.	Position(bp)	**XPEHH (F_{ST}) Scores**	Trait, Direction	QTL Name
1	75034459-75999912	0.34<0.35<0.73 (0.06<0.07<0.12)	BF6, Low	Intermuscular fat content QTL；
1	270659255-271411666	0.44<0.59<0.80 (0.06<0.09<0.13)	BF1, Low	Drip loss QTL；
2	119980473-120922605	0.44<0.57<0.62 (0.05<0.12<0.23)	BF1, Low	Average daily gain QTL；
6	40436890-41163330	0.33<0.44<0.79 (0.03<0.05<0.18)	BF3, Low	Fat weight (total) QTL；Belly weight QTL；Drip loss QTL；pH 45 minutes post mortem QTL；Average daily gain QTL；Loin muscle area QTL；Intramuscular fat content QTL
10	10868180-11462923	0.37<0.50<0.65 (0.08<0.16<0.18)	BF1, Low	Intramuscular fat content QTL；pH 45 minutes post mortem QTL；Triglyceride level QTL；Average backfat thickness QTL；Eicosapentaenoic acid content QTL；
14	98219317-98841746	0.28<0.35<0.57 (0.03<0.06<0.12)	BF2, Low	mummified pigs QTL；
15	115418643-116218447	0.50<0.65<0.79 (0.06<0.09<0.16)	BF1, Low	Skin thickness QTL；
12	57219422-57749302	-0.35>-0.41>-0.52 (0.07<0.10<0.20)	BF3, High	Fat weight (total) QTL; Obesity index QTL; Intramuscular fat content QTL; Marbling QTL; Muscle moisture percentage QTL; Meat color L* QTL; PH for Longissmus dorsi QTL;
13	173585408-176112123	-0.31>-0.44>-0.79 (0.04<0.05<0.13)	BF6, High	mummified pigs QTL; Body weight (birth) QTL;
15	4257226-5665872	-0.46>-0.60>-0.62 (0.05<0.10<0.13)	BF1, High	Days to 110 kg QTL; Intramuscular fat content QTL; Average backfat thickness QTL;
15	128164437-128661043	-0.50>-0.52>-0.74 (0.07<0.10<0.14)	BF1, High	Feed conversion ratio QTL
17	19479617-20272227	-0.34>-0.39>-0.61 (0.05<0.07<0.18)	BF1, High	Shear force at first peak QTL; Backfat between 3rd and 4th last ribs QTL
17	21239488-25085047	-0.37>-0.42>-0.75 (0.07<0.074<0.20)	BF2, High	Palmitic acid content QTL; Myristic acid content QTL; Eicosenoic acid to eicosanoic acid ratio QTL; Backfat between 3rd and 4th last ribs QTL

Table S7 the summary of XPEHH scores for trait-specific selection signatures.

	**BF1**	**BF2**	**BF3**	**BF4**	**BF5**	**BF6**
**Low backfat thickness subpopulation (+)**	73	59	41	58	8	48
**High backfat thickness subpopulation (-)**	53	16	9	70	9	12

Note: "+" and "-" indicated that selection signatures were detected in low and high backfat thickness subpopulations, respectively.

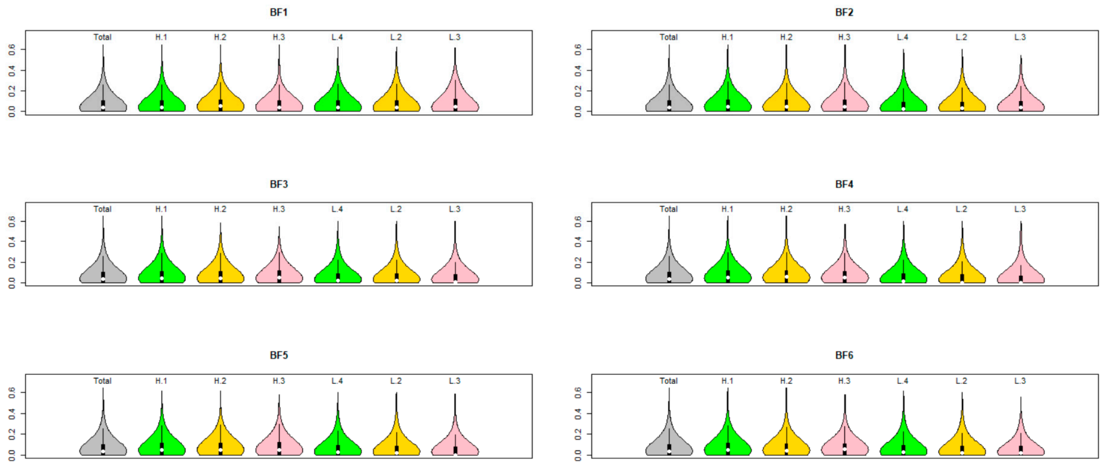

Figure S1 The violin plot of expected heterozygosity in each subpopulation for backfat thickness traits. Total represents the source Yorkshire population, H.1 (H.2, H.3) and L.1 (L.2, L.3) represents the two subpopulations of 1st (2nd, 3rd) population pair.

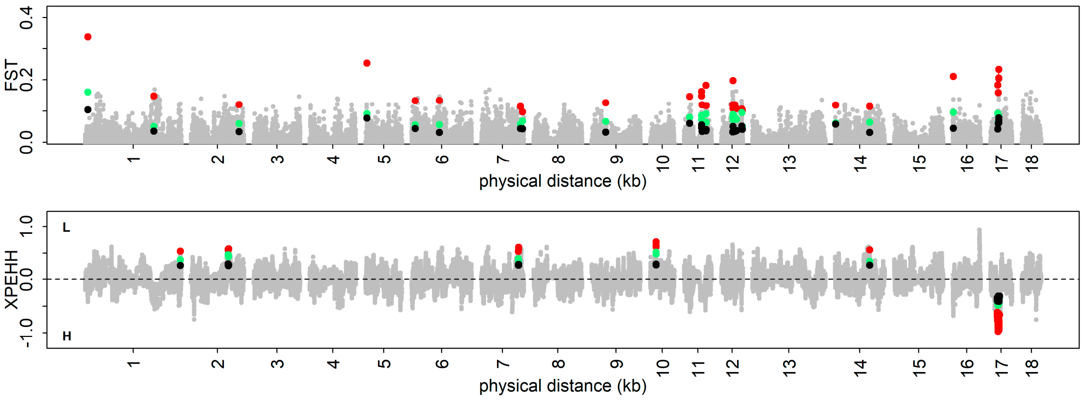

Figure S2 Visualization of trait-specific selection signatures for backfat between 6th and 7th thoracic vertebras (BF2). The colored dots indicate the trait-specific selection signatures. The black (green, red) dots indicate the selection signatures identified in 1st (2nd, 3rd) population pair.

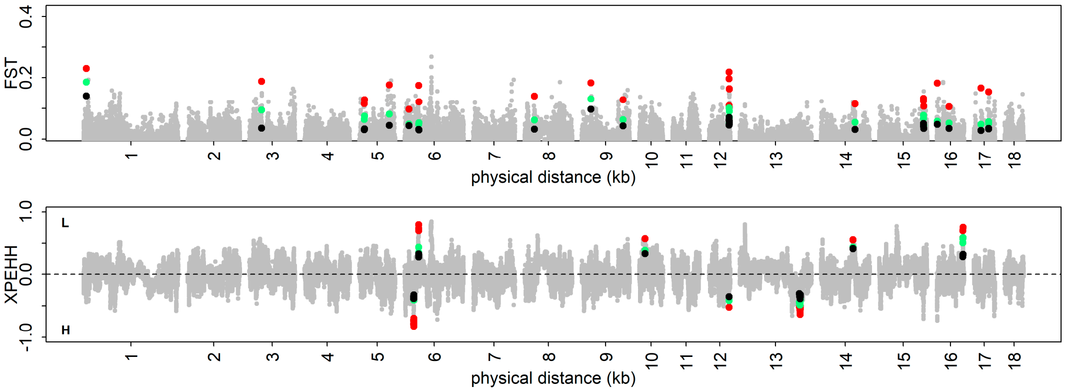

Figure S3 Visualization of trait-specific selection signatures for backfat at tenth rib (BF3). The colored dots indicate the trait-specific selection signatures. The black (green, red) dots indicate the selection signatures identified in 1st (2nd, 3rd) population pair.

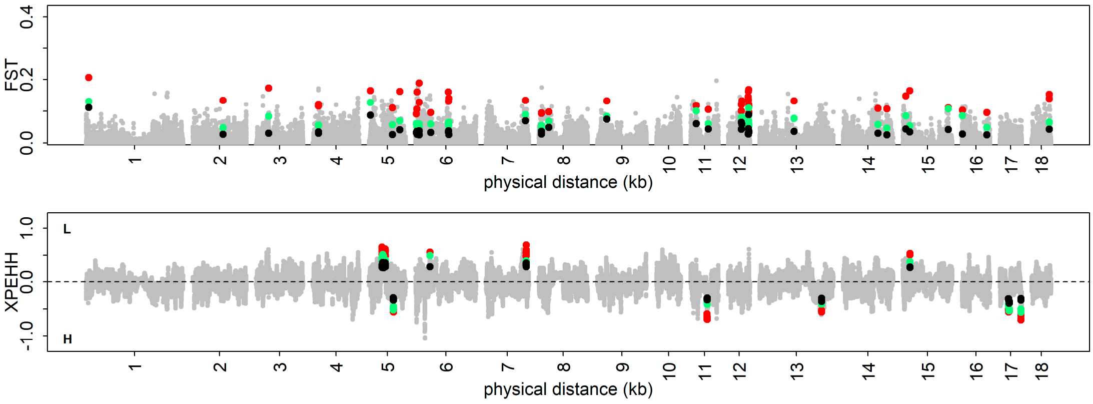

Figure S4 Visualization of trait-specific selection signatures for backfat at thoracolumbar junction (BF4). The colored dots indicate the trait-specific selection signatures. The black (green, red) dots indicate the selection signatures identified in 1st (2nd, 3rd) population pair.

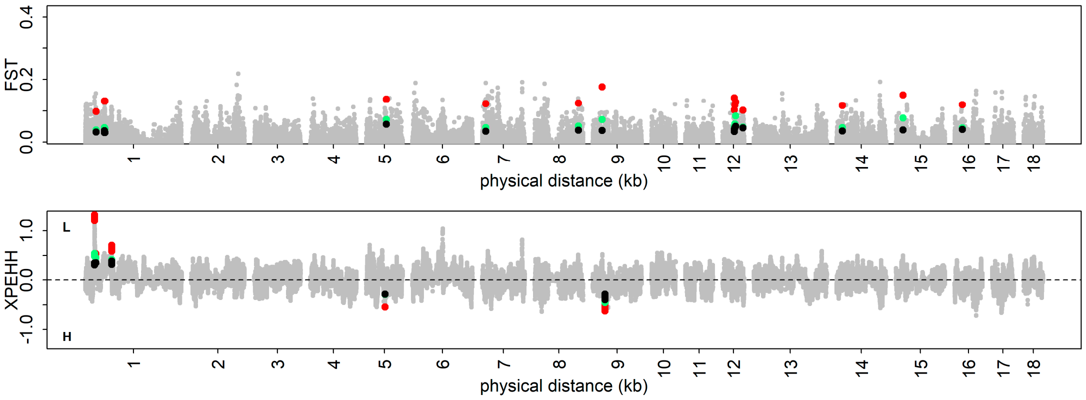

Figure S5 Visualization of trait-specific selection signatures for backfat at waist recommended vertebral junction (BF5). The colored dots indicate the trait-specific selection signatures. The black (green, red) dots indicate the selection signatures identified in 1st (2nd, 3rd) population pair.

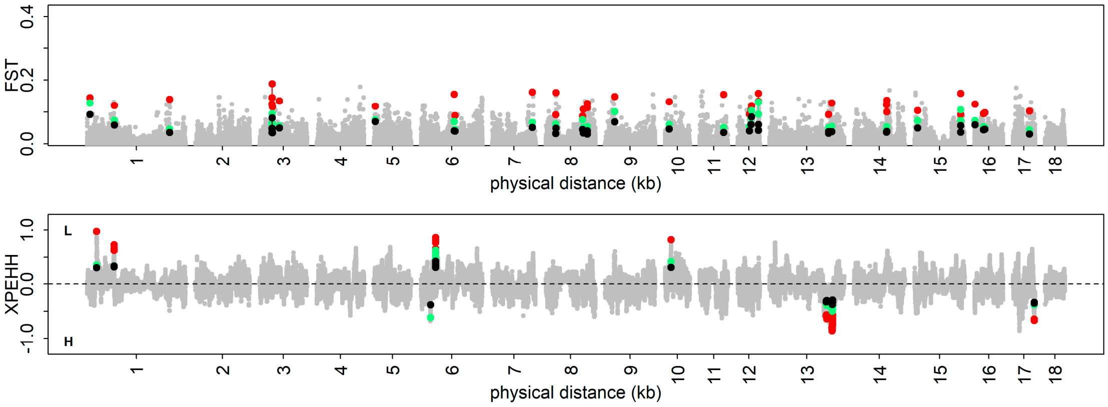

Figure S6 Visualization of trait-specific selection signatures for the average backfat thickness of 3 points (BF6). The colored dots indicate the trait-specific selection signatures. The black (green, red) dots indicate the selection signatures identified in 1st (2nd, 3rd) population pair.

References

1. Pata M, Vacher J: Ostm1 Bifunctional Roles in Osteoclast Maturation: Insights From a Mouse Model Mimicking a Human OSTM1 Mutation. *J Bone Miner Res* 2018, 33(5):888-898.
2. Suematsu N, Okamoto K, Isohashi F: Mouse cytosolic acetyl-CoA hydrolase, a novel candidate for a key enzyme involved in fat metabolism: cDNA cloning, sequencing and functional expression. *Acta Biochim Pol* 2002, 49(4):937-945.
3. Mardy S, Miura Y, Endo F, Matsuda I, Sztriha L, Frossard P, Moosa A, Ismail EAR, Macaya A, Andria G *et al*: Congenital insensitivity to pain with anhidrosis: Novel mutations in the TRKA (NTRK1) gene encoding a high-affinity receptor for nerve growth factor. *Am J Hum Genet* 1999, 64(6):1570-1579.
4. Dong B, Vold S, Olvera-Jaramillo C, Chang H: Functional redundancy of frizzled 3 and frizzled 6 in planar cell polarity control of mouse hair follicles. *Development* 2018, 145(19).
5. Ropka-Molik K, Eckert R, Piorkowska K: The expression pattern of myogenic regulatory factors MyoD, Myf6 and Pax7 in postnatal porcine skeletal muscles. *Gene Expr Patterns* 2011, 11(1-2):79-83.
6. Kim KS, Lee JJ, Shin HY, Choi BH, Lee CK, Kim JJ, Cho BW, Kim TH: Association of melanocortin 4 receptor (MC4R) and high mobility group AT-hook 1 (HMGA1) polymorphisms with pig growth and fat deposition traits. *Anim Genet* 2006, 37(4):419-421.
7. Chen QL, Quan C, Xie BX, Chen L, Zhou SL, Toth R, Campbell DG, Lu SS, Shirakawa R, Horiuchi H *et al*: GARNL1, a major RalGAP alpha subunit in skeletal muscle, regulates insulin-stimulated RalA activation and GLUT4 trafficking via interaction with 14-3-3 proteins. *Cell Signal* 2014, 26(8):1636-1648.
8. Zhang QH, Seo S, Bugge K, Stone EM, Sheffield VC: BBS proteins interact genetically with the IFT pathway to influence SHH-related phenotypes. *Hum Mol Genet* 2012, 21(9):1945-1953.
9. Lao O, de Gruijter JM, van Duijn K, Navarro A, Kayser M: Signatures of positive selection in genes associated with human skin pigmentation as revealed from analyses of single nucleotide polymorphisms. *Ann Hum Genet* 2007, 71:354-369.
10. Xu HP, He XM, Fang MX, Hu YS, Jia XZ, Nie QH, Zhang XQ: Molecular cloning, expression and variation analyses of the dopamine D2 receptor gene in pig breeds in China. *Genet Mol Res* 2011, 10(4):3371-3384.
11. Massuto DA, Kneese EC, Johnson GA, Burghardt RC, Hooper RN, Ing NH, Jaeger LA: Transforming growth factor beta (TGFB) signaling is activated during porcine implantation: proposed role for latency-associated peptide interactions with integrins at the conceptus-maternal interface. *Reproduction* 2010, 139(2):465-478.
